# Hepatic arterial embolization procedures in neuroendocrine tumors with carcinoid heart disease: a retrospective single-center study on safety and feasibility

**DOI:** 10.1530/ERC-26-0128

**Published:** 2026-07-27

**Authors:** Théodore Vuong, Lambros Tselikas, Julien Hadoux, Sondés Smaali, Matthieu Faron, Thierry De Baère, Livia Lamartina, Sophie Moog, Elena Fernandez De Sevilla, Paul Beunon, Stéphane Ederhy, Yves Menu, Frédéric Deschamps, Éric Baudin, Baptiste Bonnet

**Affiliations:** ^1^ Département d’Anesthésie Chirurgie et Interventionnel (DACI), Service d’Imagerie Thérapeutique, Gustave Roussy, Villejuif, France; ^2^ Faculté de Médecine, Université Paris-Saclay, Le Kremlin-Bicêtre, France; ^3^ Département de Médecine Oncologique, Gustave Roussy, Villejuif, France; ^4^ Institut d’Oncologie Thoracique, Hôpital Marie Lannelongue, Le Plessis-Robinson, France; ^5^ Département d’Anesthésie Chirurgie et Interventionnel (DACI), Service de Chirurgie Digestive, Gustave Roussy, Villejuif, France; ^6^ Service de Cardiologie, Hôpital Pitié-Salpêtrière, Paris, France; ^7^ Département d’Imagerie Médicale, Service de Radiologie Diagnostique, Gustave Roussy, Villejuif, France

**Keywords:** carcinoid heart disease, hepatic arterial embolization, neuroendocrine tumor, carcinoid syndrome, safety

## Abstract

Hepatic arterial embolization reduces liver tumor burden and hormonal secretion in neuroendocrine tumors, but its safety in patients with carcinoid heart disease is underexplored, and current guidelines suggest managing the cardiac disease first. We assessed the safety and efficacy of hepatic arterial embolization in patients with neuroendocrine tumors and concomitant carcinoid heart disease in a single-center retrospective study including patients with liver metastases and carcinoid heart disease confirmed on expert transthoracic echocardiography between 2001 and 2025. The primary endpoint was procedural safety; secondary endpoints were symptom control, biochemical response, and overall survival. Twenty-seven patients (median age: 58.6 years) underwent 69 embolization procedures using bland, conventional, and drug-eluting bead techniques. Severe carcinoid heart disease was present in 52% of patients, and right-sided valvular disease was found in 96%, with tricuspid regurgitation predominating. No procedure-related or cardiovascular death occurred. Two cardiac complications (7%) arose, both in patients with severe pre-existing valvular disease, together with four extra-cardiac complications. Carcinoid syndrome symptoms and urinary 5-hydroxyindoleacetic acid levels decreased significantly after treatment, whereas cardiac symptoms remained stable. The median overall survival was 6.25 years, with a 5-year survival of 60%. Hepatic arterial embolization appears feasible with an acceptable safety profile in patients with neuroendocrine tumors and concomitant carcinoid heart disease when performed in expert centers with multidisciplinary management and may contribute to carcinoid syndrome control. Prospective multicenter studies are warranted to confirm these findings and define the optimal sequencing of hepatic arterial embolization and cardiac valve surgery.

**Key findings:**

– Safety of HAE in carcinoid heart disease:
  Hepatic arterial embolization demonstrated a favorable safety profile in NET patients with mild to moderate CHD, with no cardiac-related deaths. However, cardiac complications occurred exclusively in patients with severe CHD (14.3%), underscoring the importance of careful patient selection and expert multidisciplinary management in this subgroup.
– Significant improvement in carcinoid syndrome symptoms:
  HAE was associated with a marked and statistically significant reduction in carcinoid syndrome symptoms, notably diarrhea and flushing, as well as a significant decrease in 24-h u5-HIAA levels, underscoring its antisecretory potential even in this high-risk population.
– HAE as a potential bridge to cardiac surgery:
  Reducing hepatic tumor burden through HAE prior to valve replacement may help prepare patients for surgery and potentially lower the risk of bioprosthetic valve degeneration. Prospective multicenter studies with standardized protocols are needed to confirm these findings and define the optimal therapeutic sequencing in this population.

## Introduction

Neuroendocrine tumors (NETs) represent a heterogeneous group of rare neoplasms arising from neuroendocrine cells distributed throughout the body, with the gastrointestinal tract and lungs being the most common primary sites (1, 2, 3, 4, 5). These tumors are characterized by considerable biological diversity, ranging from indolent, well-differentiated low-grade lesions to aggressive, poorly differentiated neuroendocrine carcinomas (6, 7). Well-differentiated NETs typically exhibit a slow, indolent clinical course and are associated with a relatively favorable prognosis, even in the setting of metastatic disease. Population-based studies and large registry analyses have consistently demonstrated a median overall survival exceeding 5 years in patients with metastatic well-differentiated NETs, with 5-year survival rates ranging from 38 to 52% depending on tumor grade and primary site (8, 9, 10).

Over recent decades, the incidence and prevalence of NETs have risen substantially, driven by increased clinical awareness, widespread use of cross-sectional imaging, and improved endoscopic and histopathological detection methods (11). This trend has been particularly pronounced for localized and well-differentiated tumors, reflecting earlier diagnosis of previously undetected indolent disease (4, 10). A defining feature of NETs is their capacity for hormone secretion, giving rise to functional syndromes that significantly impact clinical presentation and patient outcomes (2, 7). Among these, carcinoid syndrome is the most common, occurring in approximately 20% of patients with NETs overall and up to 50% in those with metastatic midgut tumors (12). The syndrome is characterized by episodic flushing and diarrhea, driven predominantly by excessive serotonin secretion, reflected biochemically by elevated urinary 5-hydroxyindoleacetic acid (5-HIAA). While serotonin is the principal mediator, evidence also supports a contributory role for tachykinins and catecholamines, whereas the involvement of histamine, bradykinin, and prostaglandins remains insufficiently substantiated (13).

Carcinoid syndrome substantially impairs quality of life and is associated with serious long-term complications, most notably carcinoid heart disease (CHD) (2, 12). CHD develops in 20–50% of patients with carcinoid syndrome and results from chronic exposure to elevated circulating serotonin, leading to fibrotic plaque-like deposits on the endocardium (14). These deposits predominantly affect the tricuspid and pulmonary valves (in more than 90% of cases), resulting in tricuspid regurgitation and/or pulmonary valve stenosis, which lead to progressive right ventricular dysfunction and ultimately congestive heart failure (15). Surgical valve replacement, typically involving both the tricuspid and pulmonary valves, remains the only definitive treatment for advanced CHD and is associated with a marked symptomatic improvement and an improved survival compared to medical management alone (16). CHD represents a major independent negative prognostic factor, with recent data demonstrating a nearly two-fold to three-fold increase in mortality risk and median overall survival of approximately 3–5 years from CHD diagnosis (17). This underscores the critical importance of early screening and multidisciplinary management of carcinoid syndrome and its cardiac complications (18).

The European Neuroendocrine Tumor Society (ENETS) recommendations have established treatment algorithms that guide the therapeutic approach in NET patients, including dedicated recommendations for patients with functioning tumors (19, 20, 21, 22). For patients with unresectable hepatic metastases and symptoms refractory to optimal medical treatment, liver-directed loco-regional therapies should be discussed early in the multidisciplinary team (MDT) (23). Among these, hepatic arterial embolization (HAE) occupies a central role. Performed via selective catheter positioning under fluoroscopy, HAE induces tumor necrosis through occlusion of the hepatic arterial supply, a strategy that exploits the preferential arterial vascularization of hepatic metastases, in contrast to the predominantly portal supply of normal liver parenchyma (24). By reducing hepatic tumor burden, which represents the dominant site of disease in gastrointestinal NETs, HAE concurrently decreases hormonal secretion and may provide meaningful symptom control in patients with carcinoid syndrome (25). In patients not eligible for or refractory to peptide receptor radionuclide therapy (PRRT) (26, 27), HAE represents a key therapeutic option, although patient selection must carefully account for hepatic function, tumor burden, and comorbidities.

The coexistence of CHD with advanced NET presenting hepatic metastases amenable to HAE represents a particularly challenging clinical scenario. ENETS guidelines suggest that loco-regional therapies may be relatively contraindicated in the setting of CHD and recommend managing cardiac disease prior to considering such interventions (28). This recommendation is grounded in the hemodynamic risks inherent to the procedure: HAE can precipitate a carcinoid crisis, a life-threatening surge in vasoactive mediators, resulting in acute hemodynamic instability that may be particularly poorly tolerated in patients with pre-existing right ventricular dysfunction and reduced cardiac reserve (29). In addition, the post-embolization syndrome, characterized by transient systemic inflammation and fluid shifts, may further compromise cardiac function in this vulnerable population (30). Despite these concerns, no dedicated study has systematically assessed the safety of HAE in patients with concomitant CHD, and no studies have evaluated the safety of performing cardiac valve surgery prior to HAE in this specific population.

Given this gap in the literature, our retrospective study aims to evaluate the short- and mid-term safety of HAE in patients with advanced NET and concomitant CHD. Specifically, we investigate the incidence of procedure-related complications and the impact of HAE on carcinoid syndrome and heart failure syndrome control.

## Materials and methods

### Participants

This retrospective study included all patients with NET and CHD as defined by transthoracic echocardiogram (TTE) (31) who underwent interventional radiology treatment for NET-liver metastases (NETLM) at our tertiary center between 2001 and 2025. The inclusion criteria were as follows: i) a pathologically confirmed diagnosis of NETLM according to WHO (World Health Organization) and UICC TNM classifications (32, 33), ii) CHD diagnosed by an expert cardiologist working in a NET expert center certified by national and European reference networks on standardized TTE criteria, iii) availability of comprehensive radiological reports detailing procedures performed and follow-up, and iv) age > 18 years. The exclusion criteria were as follows: i) interventional radiology treatment other than HAE, ii) absence of CHD on expert TTE report, iii) HAE performed before CHD diagnosis and no HAE performed after, and iv) patients lost to follow-up. Patients for whom no expert TTE report confirming CHD was available were analyzed separately in the complementary carcinoid syndrome (CS) cohort based on clinical symptoms. CHD cardiac surgery was not a contraindication for HAE.

Clinical data, including demographics, clinical history, tumor grade, and prior therapies, were extracted from patient records, focusing on carcinoid syndrome symptoms (diarrhea, flushing, and bronchospasm), cardiac symptoms (CHF, edema, and jugular distension), and OS, defined as the time from the diagnosis of CHD (by TTE) to death. Data on carcinoid syndrome-related symptoms, specifically flushing and diarrhea (expressed as the mean number of episodes per day), were retrospectively extracted from clinical consultation records at baseline and at each follow-up time point after HAE, in the context of optimal medical management per the guidelines applicable at the time of treatment, including somatostatin analog therapy. No standardized patient-reported outcome instrument was used for symptom quantification. Treatment tolerance and adverse events were graded according to the Common Terminology Criteria for Adverse Events (CTCAE V5, 2017) (34). Biochemical data, particularly 24-h urinary 5-HIAA (24-h u5-HIAA) assays, within 3 months prior to and 6 months after HAE, were extracted from patient records when available. Hepatic tumor burden was estimated by visual assessment of cross-sectional imaging (CT or MRI) by the interventional radiologist and classified as high (>50%) or low (≤50%).

TTE was performed in all patients with carcinoid syndrome, and systematically prior to any HAE procedure. Cardiac assessment focused primarily on tricuspid and pulmonary valve morphology and function, as these are the valves predominantly affected in CHD; cardiac MRI was not performed as part of the evaluation protocol. TTE acquisitions were reviewed in a blinded fashion against clinical data by two expert cardiologists, included in the MDT of our tertiary center, and graded according to ENETS criteria (31) as mild (grade I), moderate (grade II), or severe (grade III) valve dysfunction (35, 36). Notably, biological markers including 24-h u5-HIAA and NT-proBNP levels were not incorporated into the CHD grading classification, which was based exclusively on echocardiographic findings. After HAE, TTE was performed every 6 months or annually, depending on the severity of CHD. Cardiology consultations were reviewed to assess for clinical evidence of right-sided heart failure, including peripheral edema, jugular venous distension, and hepatojugular reflux, as well as left-sided heart failure signs when present.

### Hepatic arterial embolization (HAE)

An HAE session was defined as a procedure targeting a specific region of the liver (lobe, segment, or sector). An HAE cycle encompassed all sessions required to treat the entirety of known detectable liver metastases comprehensively. Sessions were spaced at intervals of 4–6 weeks, allowing for individualized assessment of therapeutic response and hepatic reserve.

Given the long duration of this cohort (>20 years), various HAE techniques were employed, adapting to medical progress (37, 38). For all HAE techniques, embolization endpoints were defined as proximal occlusion, characterized by a persistent contrast stasis within the tumor-feeding arteries for at least three cardiac cycles.

Periprocedural octreotide prophylaxis was systematically administered from 2010 onward and was, therefore, given to 21 out of 27 patients (78%). The six patients treated before 2010 did not receive periprocedural octreotide. The protocol consisted of an intravenous bolus of 250–500 μg of octreotide administered 30 min to 1 h before the start of the procedure, followed by a continuous intravenous infusion of 50–500 μg/h maintained throughout the procedure and for 24–48 h afterward, adjusted according to individual patient risk. An additional rescue intravenous bolus of 250–500 μg was available in the event of intraoperative signs of carcinoid crisis (28, 29).

Bland embolization, without chemotherapy, was performed using ethiodol oil (Lipiodol®) or resorbable gelatinous agent (Gelistapon®) in two patients deemed unfit for chemotherapeutic agents.

Conventional hepatic transarterial chemoembolization (c-TACE) was performed using a cytotoxic agent: doxorubicin (50–100 mg), idarubicin (5–10 mg), or oxaliplatin (50–100 mg) emulsified in 10–15 mL of ethiodol oil (Lipiodol®, Guerbet, France). The agent choice and dose were at the discretion of the interventional radiologist, considering patient medical history, prior treatments, and comorbidities. From 2013 onward, doxorubicin was abandoned based on the results of phase II and phase III trials (39, 40). After achieving distal occlusion of the tumor-feeding arteries, additional proximal embolization was performed using 1–3 mm resorbable gelatinous agents (Gelitaspon®; Gelita Medical, The Netherlands) or 300–500 μm non-resorbable beads (EmboGold® or Embospheres®; Merit Medical Systems Inc., USA) (41).

Drug-eluting bead transarterial chemoembolization (DEB-TACE) was performed using DC-BEADS® (Biocompatibles, UK) preloaded with a concentration of 25 mg/mL doxorubicin. Up to two vials (each containing 2 mL of beads) were used per session, in sizes ranging from 100 to 700 μm, selected at the discretion of the operator. Beads were diluted in a 1:1 mixture of iodinated contrast medium and saline, as per guidelines (42).

All HAE indications were validated during dedicated multidisciplinary board meetings, involving both diagnostic and interventional radiologists.

HAE was indicated in the following situations: i) diffuse or progressive unresectable liver metastases with an expected >50% tumor burden treatable; ii) uncontrolled hormone-related symptoms despite optimized medical therapy.

### Endpoints

#### Primary endpoint

The primary endpoint was procedure safety, reflected by the incidence of HAE-related adverse events, with a specific focus on cardiovascular complications. Only adverse events exceeding the expected CTCAE grade 1 post-embolization syndrome (e.g. mild abdominal pain, nausea, fever, and transient liver enzyme elevation) were reported (43, 44). Adverse events occurring within 24 h of HAE were recorded as immediate post-procedural complications, while those occurring beyond 24 h were recorded as delayed complications.

Cardiovascular adverse events included CHF, acute pulmonary edema, arrhythmias, right-sided heart failure, left-sided heart failure, and deaths from cardiovascular origin.

Non-cardiac adverse events included all other procedure-related complications whether or not they resulted in prolonged hospitalization.

A prolonged hospitalization was defined as hospitalization exceeding 4 days (45).

#### Secondary endpoints

Secondary endpoints included procedure clinical efficacy, assessed by the evolution of carcinoid and cardiac symptoms following HAE, OS, TTE, and biochemical response, evaluated through 24-h urinary 5-HIAA levels pre- and 6 months after HAE when available (46, 47).

Clinical follow-up was performed according to standard guidelines with a post-HAE consultation by the interventional radiologist at 6 weeks, followed by alternating follow-up between the oncologist and the interventional radiologist.

#### Additional analysis

In an additional analysis, we extended the cohort to include patients in whom CHD was mentioned in the medical records (carcinoid syndrome cohort (CS cohort)), but for whom an expert TTE report formally confirming the diagnosis was not available. This broader cohort, based on less stringent inclusion criteria, allowed us to further assess the primary endpoint of safety and OS.

#### Statistical analysis

All statistical analyses were performed using R software, version 4.4.2 (The R Core Team, 2024, Austria). Given the retrospective design and the paired nature of the data, analyses were conducted on matched data. For categorical outcomes, the McNemar test with continuity correction was applied. For ordinal data that do not follow a normal distribution according to the Shapiro–Wilk test, the non-parametric Wilcoxon signed-rank test was used to compare before and after HAE values. A Fisher’s exact test was used to compare the proportion of complications between severe and non-severe patients. The survival curve was calculated according to the Kaplan–Meier method. To account for the relatively small sample size, complication rate was compared to a theoretical threshold of 5% using an exact binomial test. A two-tailed *P*-value ≤0.05 was considered statistically significant for all analyses.

This study was conducted in compliance with the principles of the Declaration of Helsinki (2013) and received approval from our institutional ethics committee and review board (IRB No. 2025-544). Given the retrospective nature of the study, patients received a letter of non-opposition regarding the use of their data for scientific research purposes.

## Results

### Participants’ characteristics

Of the 105 patients initially identified as having neuroendocrine tumors (NETs) and carcinoid syndrome who had undergone interventional radiology treatment, 27 met all the inclusion criteria and were included in the final analysis (13 females and 14 males). Thirty-six patients were excluded from the study due to having undergone interventional radiology treatments other than HAE. Eighteen patients were excluded because echocardiographic evaluation did not confirm the presence of CHD. Seventeen patients were excluded because they received HAE before CHD diagnosis and did not receive HAE after. One patient was lost to follow-up.

**Figure 1:**
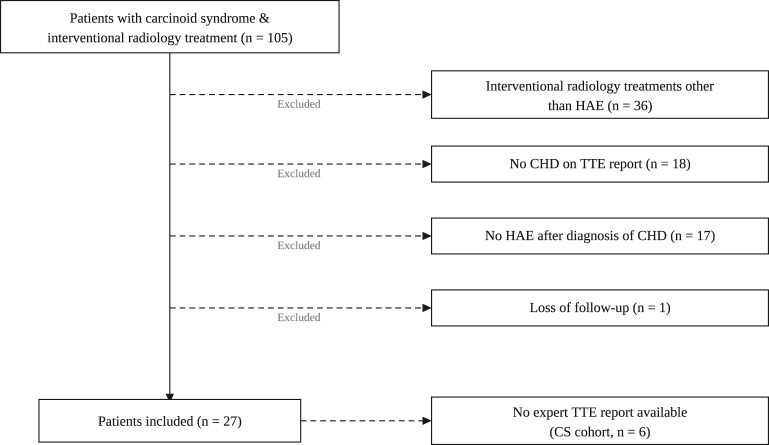
Flow chart. HAE: hepatic arterial embolization, TTE: transthoracic echocardiography, CHD: carcinoid heart disease.

Patients for whom no expert TTE report confirming CHD was available were analyzed separately in the CS cohort (*n* = 6). The flow chart of patient selection is presented in Fig. 1.

Most patients had advanced ileal NETs (*n* = 24, 89%). The median age was 58.6 years (IQR: 49–69 years). High hepatic tumor burden (>50%) was observed in 18 patients (67%). Extrahepatic metastases were predominantly peritoneal (*n* = 17, 63%). Carcinoid syndrome, at the time of HAE, included diarrhea (*n* = 24, 89%) and/or flushing (*n* = 22, 81%) without bronchospasm. At baseline, before HAE, 6 patients (22%) were symptomatic with both left- and right-sided signs of heart failure, 2 patients were symptomatic with isolated left-sided symptoms, such as dyspnea (7%), and 3 patients with isolated right-sided symptoms, such as edema and jugular distension (11%). Sixteen patients (59%) had available 24-h u5-HIAA levels before and after HAE (median time: 6 months). Full patient characteristics are summarized in Table 1.

**Table 1:** Patients’ characteristics.

Sex, no. (%)	
– Male	14 (52%)
– Female	13 (48%)
Age, year (median, IQR)	58.6 (49–69)
NET type, no. (%)	
– Ileum	24 (89%)
– Colon–rectum	1 (4%)
– Pulmonary	2 (7%)
Tumor grade, no. (%)	
– G1	11 (41%)
– G2	10 (37%)
– G3	2 (7%)
– NA	4 (15%)
Site of metastases at first HAE, no. (%)	
– Liver only	5 (19%)
– Liver + others	22 (81%)
○Peritoneum	17 (63%)
○Bone	6 (22%)
○Mediastinal	2 (7%) (pulmonary tumor)
○Lymph node	1 (3.7%)
○Ovary	1 (3.7%)
○Pancreas	1 (3.7%)
○Adrenal gland	1 (3.7%)
○Spleen	1 (3.7%)
High hepatic tumor burden, no. (%)	18 (67%)
Carcinoid-related syndrome, no. (%)	
– Diarrhea	24 (89%)
– Flushing	22 (81%)
Carcinoid-related symptoms, mean per day (IQR)	
– Diarrhea	3.4 (1–5)
– Flushing	3.3 (1–4)
Cardiac symptoms, no. (%)	11 (41%)
– Left-sided sign (dyspnea)	2 (7%)
– Right-sided sign (edema, jugular turgidity)	3 (11%)
– Both left- and right-sided signs	6 (22%)
24-h u5-HIAA, median xULN (IQR)	7.3 (3.6–10.3)

NET, neuroendocrine tumor; HAE, hepatic arterial embolization; xULN, x-times the upper limit of normal. *Some patients were metastatic at multiple sites*.

Regarding cardiac involvement at TTE, most patients (*n* = 26, 96%) had right-sided valvular disease, with tricuspid regurgitation identified in 96% of patients and pulmonary stenosis in 41%. Among those, 10 patients (37%) had concomitant left-sided CHD. Only one patient (3%) with lung primary had isolated left-sided CHD. CHD severity was classified as severe in 52% (*n* = 14), moderate in 22% (*n* = 6), and mild in 19% (*n* = 5). No consensus on severity grading was reached between the two expert cardiologists for the remaining two patients (7%); as these patients were not classified as severe, they were grouped with the mild and moderate cases in the non-severe group (*n* = 13, 48%) used for the safety analysis. The mean grade of the predominant valve regurgitation, defined as the most severe valve lesion when both valves were affected, was 2.36 (±0.8). Right ventricular dysfunction was identified in 26% at TTE (*n* = 7).

Concerning CHD cardiac surgery, three patients (11%) were treated by valve replacement prior to HAE, with a median time between cardiac surgery and HAE of 11.6 months.

Tumor and functioning syndrome-related prior treatments are reported in Supplemental Table 1 (see section on Supplementary materials given at the end of the article). All patients received somatostatin analog treatment prior to and/or concomitant with HAE, in accordance with the recommendations applicable at the time of treatment.

### HAE procedures

**Figure 2:**
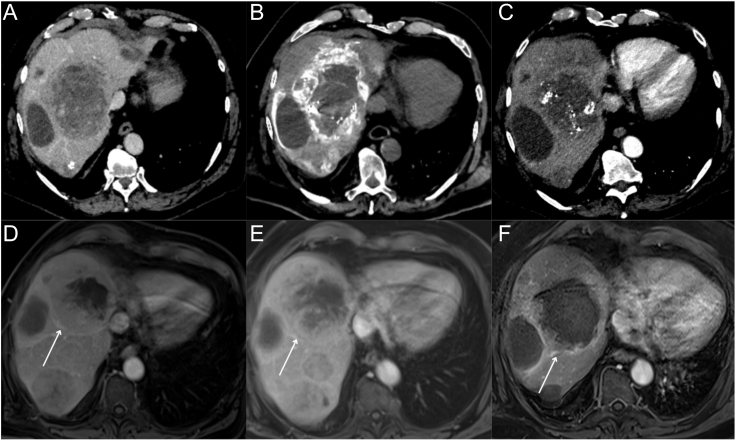
Pre- and post-operative imaging of a c-TACE procedure in a 70-year-old patient with pulmonary NET. (A) The CT scan of the liver in axial plane at portal phase 6 weeks before c-TACE procedure shows a voluminous heterogeneous metastasis of the hepatic dome and a scar of a lesion previously treated in segment VIII. (B) Preoperative cone-beam CT reveals peripheral arterial enhancement of the targeted metastasis with a devascularized center. (C) The follow-up CT scan of the liver in axial plane at arterial phase at 5 months shows a complete peripheral devascularization of the targeted metastasis, with the presence of intra-tumoral Lipiodol. (D) MRI in axial plane, T1-weighted image at arterial phase after gadolinium injection 4 weeks before c-TACE, showing the fleshy portion of the metastasis. (E) The follow-up MRI in axial plane, T1-weighted image at arterial phase at one month reveals a partial devascularization. (F) The follow-up MRI in axial plane, T1-weighted image at arterial phase at 6 months shows a complete devascularization.

A total of 69 HAE procedures were performed among 27 patients (Fig. 2), consisting of 55 c-TACE (80%), 12 DEB-TACE (17%), and 2 bland TAE (3%). The median interval between NET diagnosis and the first HAE session was 868 days (IQR: 184–1,561).

Among all HAE procedures, 28 (41%) were performed in patients with severe CHD and 41 (59%) in the non-severe group.

The distribution of HAE sessions varied: 18 patients (67%) underwent a single HAE cycle (1–3 sessions), while 9 patients (33%) underwent 2–4 complete cycles of HAE. The mean number of HAE procedures per patient was lower in the severe CHD subgroup compared to the non-severe CHD subgroup (2.0 vs 3.8, respectively).

### Primary endpoint

Among the 27 patients and 69 HAE procedures, the median hospital stay was 4 days (IQR: 3–5 days). No procedure-related death was reported. Six complications exceeded grade 1 post-HAE syndrome. These complications occurred within 1–42 days after HAE and were classified as cardiac (*n* = 2, 7%) or extra-cardiac (*n* = 4, 15%) events including one carcinoid syndrome worsening.

Regarding the two cardiac complications, one severe case (CTCAE grade 3) involved acute pulmonary edema with renal, hepatic, and mild neurological impairment (asterixis and mild encephalopathy), which occurred one day post-procedure, requiring prolonged hospitalization for 14 days. The patient recovered fully with specialized care in a cardiac intensive care unit. The second cardiac event (CTCAE grade 2) occurred 3 days post-procedure, manifested as mild left heart failure managed effectively with diuretics and without prolonged hospitalization. Notably, cardiac complications occurred in 2 out of 14 patients with severe CHD (14.3%), corresponding to 2 of the 28 HAE procedures performed in this subgroup, while no complications were observed in the 13 patients in the non-severe group (*P* = 0.16). No additional cardiac adverse events were reported during the one-year evaluation after the HAE procedure nor was there any worsening of the CHD grading within the year.

Regarding hepatic tumor burden, five of the six reported complications exceeding grade 1 post-HAE syndrome occurred in patients with high hepatic tumor burden (>50%). These included both cardiac complications and three of the four extra-cardiac complications (hepatic encephalopathy, general condition deterioration with carcinoid crisis worsening, and general condition deterioration managed with nutritional support). The only complication observed in a patient with low hepatic tumor burden (≤50%) was a pseudoaneurysm at the puncture site resolved with manual compression. These findings suggest that high hepatic tumor burden may represent an additional risk factor for procedure-related complications, independent of CHD severity.

Among the extra-cardiac complications, all were procedure related. One severe case (CTCAE 3) involved hepatic insufficiency with hepatic encephalopathy, which resolved favorably in 3 days, without extending hospital stay. A second severe complication was a deterioration in general condition including a carcinoid syndrome worsening of a patient already hospitalized in intensive care for carcinoid crisis (CTCAE 4). Two were non-severe complications (CTCAE 2): i) a pseudoaneurysm at the puncture site that resolved with manual compression and ii) a deterioration in general condition successfully managed with nutritional support. Extra-cardiac complications were not found to be related to the severity of CHD (*P* = 0.60).

### Secondary endpoints

Follow-up of carcinoid symptoms showed a significant improvement in both diarrhea (from a mean of 3.4 stools per day to 0.4, *P* < 0.0001) and flushing (from a mean of 3.3 episodes per day to 0.6, *P* < 0.0001). However, no statistical improvement was observed in cardiac symptoms, although a trend toward a reduction in symptoms was noted (Table 2).

**Table 2:** Comparison of carcinoid and heart failure symptoms before and after HAE. Carcinoid syndrome symptoms are expressed as the mean number of episodes per day, and heart failure signs are expressed as the number of patients presenting the symptom.

Type of symptom	Pre-HAE	Post-HAE	*P*-value
Carcinoid syndrome (mean per day)			
Diarrhea	3.4	0.4	<0.0001
Flushing	3.3	0.6	<0.0001
Heart failure (no. patients)			
Dyspnea	8	4	0.22
Edema	9	4	0.06
Jugular turgidity	6	1	0.07

Two out of 27 patients required valve replacement surgery during post-HAE follow-up. One patient progressed in CHD from mild to severe over 2 years after the last HAE, together with increased hormonal secretion and tumor progression. This patient successfully underwent bioprosthetic valve replacement, followed by a second cycle of c-TACE 3 months after cardiac surgery, with no complications reported over a 5-year post-operative follow-up. Another patient who had planned valve replacement before HAE underwent a subsequent cycle of HAE post-surgery, with no additional complications. Among patients with prior valve surgery, subsequent follow-up showed no deterioration of CHD.

**Figure 3:**
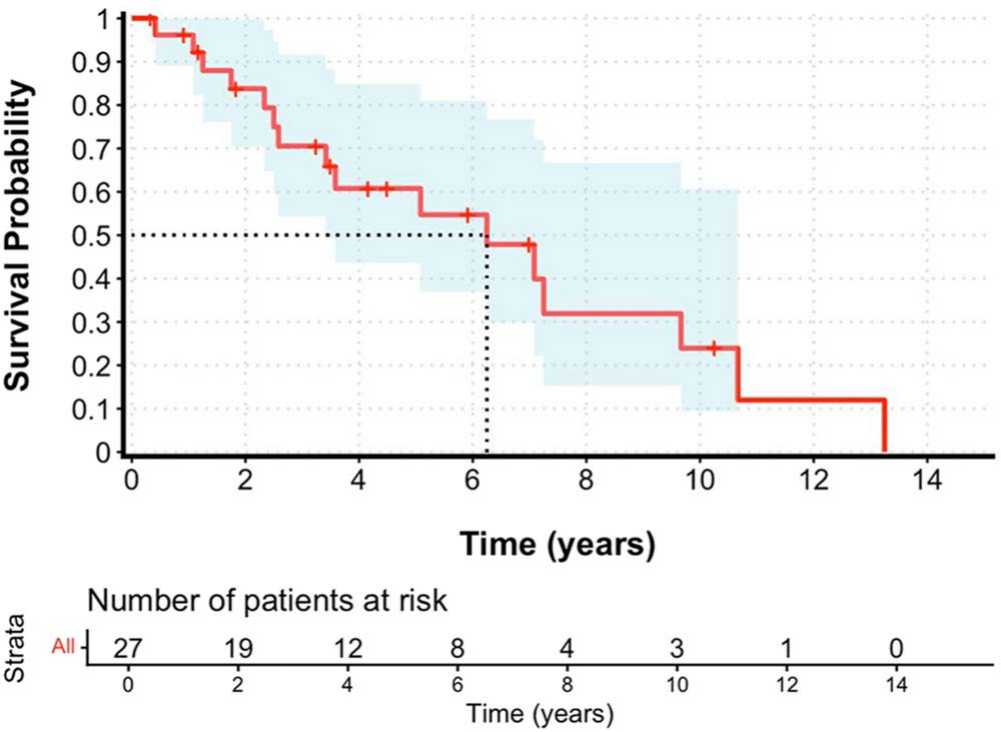
Kaplan–Meier curve estimating overall survival (OS) with the first TTE of CHD considered as the baseline. The median OS is 6.25 years.

After a median follow-up of 5 years, OS analysis showed a median survival of 6.25 years. Estimated survival rates were 96% at 1 year, 70% at 3 years, and 60% at 5 years. Of the 27 patients, 17 had died and 10 were censored (Fig. 3).

**Figure 4:**
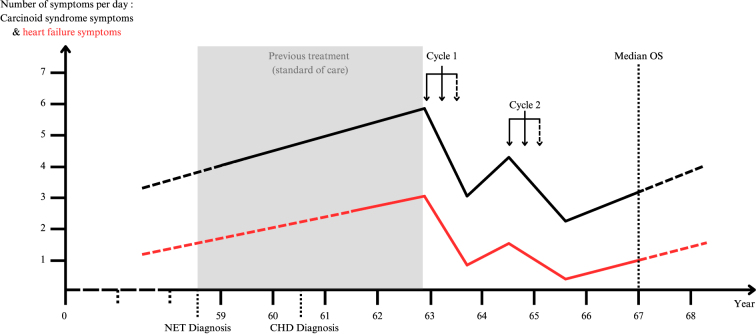
Modeling of the average symptomatic evolution of patients. The black line represents the number of carcinoid symptoms per day. The red line indicates the number of cardiac symptoms (congestive heart failure, edema, and jugular distension) per patient.

Figure 4 reports the evolution of carcinoid and cardiac-related symptoms during the treatment period.

During follow-up, valvular dysfunction grade remained stable at 1 year after HAE. Right ventricular function appeared to improve following the procedure, although this trend did not reach statistical significance, likely owing to the limited sample size.

Regarding biomarkers, among the 16 patients with available paired measurements, the median baseline 24-h u5-HIAA level was 7.3 xULN (IQR: 3.6–10.3). Following HAE, the median 24-h u5-HIAA at 6 months decreased significantly to 3.4 xULN (IQR: 1.2–7.3, *P* = 0.006).

### Additional analysis – CS cohort

Six patients were enrolled in the CS cohort, encompassing 12 additional HAE procedures. When combined with the main cohort (27 patients, 69 HAE), the overall expanded cohort comprised 33 patients and 81 HAE procedures. The overall safety profile remained favorable: across the 33 patients and 81 HAE procedures, cardiac complications occurred in 2 patients (6%), one of which was severe, extra-cardiac complications in 4 patients (12%), and no procedure-related death was reported. The median OS was 8.1 years in the CS cohort.

## Discussion

To our knowledge, this is the first study specifically assessing the safety of HAE in patients with CHD, including 52% of severe valve dysfunction. Our study suggests the feasibility of HAE procedures prior to cardiac surgery, as evidenced by the occurrence of a severe cardiac complication in only 1 patient out of 27 (3.7%) and the absence of cardiac-related mortality. In parallel, we report a clinically significant reduction in carcinoid syndrome symptoms and 24-h u5-HIAA levels.

Out of 27 patients and 69 HAE procedures, only two cardiac complications (7%) were reported, both occurring in patients with pre-existing severe valvular disease and right ventricular dysfunction at TTE. One of these complications (CTCAE grade 3) involved multiorgan failure occurring within 24 h of the procedure, requiring admission to a specialized cardiology unit. The patient recovered fully within two weeks. The second event (CTCAE grade 2) was a milder episode of heart failure, managed effectively with diuretics without prolonged hospitalization. Notably, no cardiac complications occurred in the 13 patients in the non-severe group, suggesting a predictive role of valvular dysfunction grading classification as recently proposed by the guidelines (48). The favorable outcome of these complications reinforces the need for optimal multidisciplinary periprocedural management involving anesthesiologists, onco-endocrinologists, interventional radiologists, and specialized cardiological teams.

No cardiac or extra-cardiac complications were reported in patients treated with HAE after valve replacement. These findings suggest that carefully selected patients with CHD may safely undergo HAE procedures prior to valve surgery, although prospective validation is needed before drawing definitive conclusions regarding current management recommendations (31, 48).

The three embolization techniques employed in our cohort – bland TAE, c-TACE, and DEB-TACE – differ in their mechanisms of action and anticipated adverse effect profiles. Bland TAE induces tumor necrosis through arterial ischemia alone. Conventional TACE combines arterial occlusion with local delivery of a cytotoxic agent emulsified in Lipiodol, adding direct chemotoxicity to the ischemic effect. DEB-TACE provides sustained cytotoxic drug release from microspheres and has been specifically associated with increased hepatotoxicity in NET patients compared to Lipiodol-based techniques (38). From a cardiac standpoint, all three techniques share the risk of triggering carcinoid crisis through acute release of vasoactive mediators, while the post-embolization inflammatory syndrome may also aggravate pre-existing cardiac dysfunction. The heterogeneity of techniques and the limited numbers in each subgroup preclude any comparative safety analysis specific to patients with CHD, and conclusions regarding the relative safety of any specific technique in this population cannot be drawn from the present data.

Compared to previous large series, such as Touloupas *et al.* (25), which reported a 3% rate of severe complications among 555 HAE procedures in less complex NET patients, our results suggest a comparable safety profile even in CHD patients. Other series reported by Egger *et al.* and Kitano *et al.* (49, 50) reported complications beyond post-HAE syndrome ranging from 9.2 to 21.7%, further supporting the relative safety of HAE in expert centers even in CHD patients. Nevertheless, prospective cohorts with standardized follow-up are needed to confirm these findings.

Interestingly, the extra-cardiac complications were not related to CHD and were in line with the known risk profile of liver embolization procedures (51).

In the present cohort, no death occurred following HAE, and cardiac complications were limited and fully resolved within 14 days (maximal hospitalization stay), without sequelae at 1 year. By contrast, the reported outcomes from surgical NET-related CHD series vary, with post-operative mortality after valve replacement ranging from 6 to 19% (17, 52, 53). The causes of death in these series remain to be better specified, including the role of the carcinoid syndrome status at the time of cardiac surgery. Taken together, these results suggest that performing HAE before cardiac surgery may not pose additional preoperative safety concerns and may help to better prepare these patients for surgery by reducing the severity of carcinoid syndrome. Finally, the exploratory analysis including patients without TTE-proven CHD (CS cohort), encompassing six patients and 12 HAE procedures, did not reveal an increased post-procedural risk and median OS was consistent with existing literature (54, 55).

The efficacy of HAE in controlling carcinoid syndrome is documented in the retrospective setting mainly (23, 44, 56, 57). Our study extends this evidence to the CHD population, which will affect approximately 20–50% of carcinoid syndrome patients during their lifetime (2). In our cohort, by reducing hormone secretion, HAE significantly alleviated the carcinoid syndrome symptoms, particularly diarrhea and flushing, in patients with refractory disease despite optimal systemic therapy. A significant reduction in 24-h u5-HIAA levels was observed following treatment (58). This is in line with the study by Wängberg *et al.* (59), which demonstrated a sustained 55% reduction in 5-HIAA levels in 40 patients following HAE. This result confirms the antisecretory potential of HAE.

Although CHD is classically considered an irreversible valvular condition, our data suggest that reducing the hepatic tumor burden through HAE and the consequent decrease in circulating vasoactive mediators may slow the progression of valvular dysfunction and contribute to improvement in right-sided heart failure symptoms, including peripheral edema and jugular venous distension. Echocardiographic follow-up data available in our cohort demonstrated stability of valvular dysfunction grade after HAE, with a non-significant trend toward improvement in right ventricular function, consistent with a potential disease-modifying effect of tumor load reduction on cardiac remodeling. These findings, however, should be interpreted with caution given the retrospective nature of the study and the limited sample size.

From a cardiological perspective, an additional argument in favor of performing HAE prior to cardiac valve surgery in patients with severe CHD deserves particular emphasis. Biological prosthetic valves are susceptible to recurrent carcinoid degeneration when circulating vasoactive mediators remain elevated, potentially leading to early structural valve deterioration and the need for reintervention (16, 60). Reducing hepatic tumor burden through HAE before valve replacement may, therefore, contribute to lowering the risk of bioprosthesis degeneration by decreasing serotonin exposure to the newly implanted valves. Although our dataset does not allow direct assessment of this hypothesis given the limited number of patients who underwent valve surgery during follow-up (*n* = 2), this consideration represents a compelling rationale for the systematic multidisciplinary discussion of HAE prior to cardiac surgery in this population and warrants prospective evaluation in future studies.

While PRRT represents an established alternative for somatostatin receptor-positive NETs with hepatic metastases (26, 27), a direct safety and efficacy comparison with HAE in patients with CHD falls outside the scope of the present retrospective study. In our cohort, five patients received PRRT at some point during their treatment course, as detailed in Supplemental Table 1; receptor positivity was confirmed in these cases by treatment indication. However, DOTATATE PET/CT imaging was not systematically available throughout our >20-year enrollment period, limiting our ability to report somatostatin receptor status for all patients. Future prospective studies should address the relative safety and efficacy of HAE versus PRRT in patients with CHD, particularly regarding carcinoid syndrome control and cardiac outcomes, ideally in a randomized or controlled design.

Our study has several limitations. The retrospective, single-center design introduces inherent selection bias and limits external validity. The absence of a control cohort of NET patients with carcinoid syndrome but without CHD represents a major limitation, preventing definitive conclusions regarding the specific contribution of CHD to the observed safety outcomes after HAE. Such a comparative analysis was not feasible within the scope of this retrospective study and should be considered a priority endpoint in future prospective multicenter studies. Self-reported and retrospectively extracted symptom assessments, in the absence of a standardized patient-reported outcome instrument, may have led to subjective bias in evaluating clinical improvements. The extended patient enrollment period reflects the rarity of this condition but may also have introduced changes in clinical practice over time, particularly regarding embolization techniques and periprocedural management.

Periprocedural octreotide prophylaxis was not uniformly administered throughout the study period, having been systematically implemented only from 2010 onward; 6 of the 27 patients treated before 2010, therefore, did not receive this prophylaxis. The incomplete availability of paired 24-h u5-HIAA measurements (16 of 27 patients) reflects the retrospective design and heterogeneity of biochemical monitoring practices across the >20-year enrollment period. Somatostatin receptor status assessed by DOTATATE PET/CT was not systematically available for all patients owing to the progressive adoption of this imaging modality over the study period, precluding a comprehensive assessment of PRRT eligibility across the cohort.

Rigorous patient selection led to the exclusion of 72 patients out of the 105 initially identified, while 6 further patients were analyzed separately in the complementary CS cohort, ensuring a homogeneous main cohort but potentially introducing selection bias. The heterogeneity of embolization procedures performed, including bland TAE in 2 patients, c-TACE with three different cytotoxic agents whose selection was not protocolized, and DEB-TACE, represents a major methodological limitation. While these procedures share a common rationale based on hepatic arterial occlusion, their distinct technical characteristics and toxicity profiles preclude definitive conclusions regarding the safety of any specific technique in patients with CHD. The lower mean number of HAE performed per patient in the severe CHD subgroup (2.0 vs 3.8 in non-severe CHD) likely reflects a more cautious approach by the multidisciplinary team in patients with reduced cardiac reserve, but data on the number of planned versus performed HAE procedures were not available in our retrospective dataset.

Finally, the absence of systematic patent foramen ovale screening data and the lack of long-term echocardiographic follow-up limit the conclusions that can be drawn regarding the cardiac impact of embolization procedures in this population.

These findings should, therefore, be considered exploratory and hypothesis-generating, supporting the need for prospective multicenter studies with standardized protocols.

## Conclusion

This study provides the first dedicated evaluation of the safety of HAE procedures in patients with concomitant CHD. The heterogeneity of techniques employed, including bland TAE, c-TACE, and DEB-TACE, and the limited sample size preclude definitive conclusions regarding the safety of any specific procedure. Our findings should, therefore, be considered exploratory and hypothesis-generating, supporting the feasibility of these procedures in expert centers with multidisciplinary management, particularly in patients with mild to moderate CHD. Prospective multicenter studies with standardized protocols and homogeneous patient populations are warranted to confirm these findings and to define the optimal therapeutic sequencing, including a formal comparison with PRRT, in this challenging population.

## Supplementary materials



## Declaration of interest

The authors declare that there is no conflict of interest that could be perceived as prejudicing the impartiality of the work reported.

## Funding

This research did not receive any specific grant from any funding agency in the public, commercial, or not-for-profit sector.

## Author contribution statement

All authors were involved in conceptualization, study design, managing clinical cases, and writing, editing, and final approval of the manuscript. TV and BB conducted the statistical analysis. All authors have read and agreed to the published version of the manuscript.

## Data availability

All data generated or analyzed during this study are included in this article. Further inquiries can be directed to the corresponding author.

## Statement of ethics

This study was conducted in compliance with the principles of the Declaration of Helsinki (2013) and received approval from our institutional ethics committee and review board (IRB No. 2025-544). Given the retrospective nature of the study, patients received a letter of non-opposition regarding the use of their data for scientific research purposes.
