# Supplementary material for: Hepatic arterial embolization procedures in neuroendocrine tumors with carcinoid heart disease: a retrospective single-center study on safety and feasibility
Source: Endocr Relat Cancer. 2026 Jul 27;33(7):e260128. doi: 10.1530/ERC-26-0128 (PMC13428016; doi:10.1530/ERC-26-0128)
Supplement: Supplementary file 1 [file ERC-26-0128_supplementary_table_1.pdf]

Previous treatment	
Any previous treatment, no. (%)	27 (100%)
**Surgery**	
Primary lesion, no.	11
Secondary lesion, no.<br>- Liver metastasis (hepatectomy)<br>- Lymph nodes	4<br>3<br>1
**Radiation therapy**	
PRRT, no.<br>- 177Lu-Dotatate<br>- 90Y-Dotatoc<br>SBRT, no.	5<br>4<br>1<br>1
**Systemic therapy**	
Chemotherapy/Immunotherapy, no.<br>- 5-FU + Oxaplatin (+/- irinotecan)<br>- Platin + Etoposid<br>- 5-FU + Streptozotocin<br>- Epirubicin- Docetaxel<br>- Temozolomid<br>- Capecitabin + Bevacizumab<br>- Pembrolizumab<br>- Interferon	10<br>3<br>2<br>1<br>2<br>1<br>3<br>1<br>2
Targeted therapy (Everolimus), no.	4
Somatostatin analog, no.<br>- Somatulin<br>- Sandostatin<br>- Somatulin + Sandostatin	27<br>10<br>6<br>11

Supplemental Table. Treatment history

Previous treatments. Some patients had received multiple previous treatments.

PRRT: Peptide receptor radionuclide therapy

SBRT: stereotactic body radiation therapy
